# Focused echocardiogram by emergency physicians (EP) in resuscitation room of Accident and Emergency (A&E) Department

**DOI:** 10.1186/2036-7902-6-S1-A21

**Published:** 2014-01-31

**Authors:** YS Ong, KH Cheung, CA Graham, TH Rainer, NK Cheung

**Affiliations:** 1 Emergency Department, Prince of Wales Hospital, Shatin, Hong Kong; 2 Accident and Emergency Medicine Academic Unit, Chinese University of Hong Kong

## Background

Use of emergency echocardiogram by emergency physicians (EP) can offer great help in critical conditions.

## Objective

Description of several cases with emergency echo utilization.

## Patients and methods

Four patients with critical conditions managed in resuscitation room were described. They all underwent bedside echo by EP, yielding timely diagnosis with great impact on management.

## Results

Patient A, elderly lady with lung cancer presented with progressive dyspnoea, desaturation, and low blood pressure. Bedside echo showed dilated right ventricle and McConnell sign. Diagnosis of pulmonary embolism was confirmed by CTPA. She was given thrombolytic in A&E with hemodynamic improvement.

Patient B, 55 year old man presented with vomiting, diarrhoea, hypotension and tachycardia. Bedside echo showed severely depressed myocardial contractility. Provisional diagnosis of myocarditis was made, which proved to be the eventual diagnosis.

Patient C, 49 year-old lady presented with epigastric discomfort and desaturation. Bedside echo showed right atrial (RA) mass. CT in A&E showed large anterior mediastinal mass with invasion into RA. The diagnosis was later confirmed to be spindle cell carcinoma with RA invasion.

Patient D, 63 year-old man presented with dizziness and breathlessness. Clinically he was in profound shock, with pale appearance and cold extremities. BP was not recordable and blood gas showed severe lactic acidosis with pH 6.9. Bedside echo showed a large circumferential pericardial effusion with collapsed right chambers and plethoric IVC. Emergency pericardiocentesis was done with dramatic improvement in hemodynamic status. CT thorax showed a right lung tumour abutting the pericardium.

## Conclusion

Focused echo by emergency physicians can give early diagnosis essential to management.

